# Harmonizing Disability Data To Improve Disability Research And Policy

**DOI:** 10.1377/hlthaff.2022.00479

**Published:** 2022-10

**Authors:** Daniel Mont, Jennifer Madans, Julie D. Weeks, Heidi Ullmann

**Affiliations:** Center for Inclusive Policy, Washington, D.C.; Center for Inclusive Policy.; National Center for Health Statistics, Hyattsville, Maryland.; National Center for Health Statistics.

## Abstract

Disability is complex and multifaceted, complicating governments’ efforts to collect the high-quality, comprehensive data necessary for developing, implementing, and monitoring policies. Yet data are needed to obtain information on functioning in the population, to identify the population with disabilities, and to disaggregate indicators of well-being by disability to determine whether people with disabilities are participating in society to the same extent as those without disabilities. In this article we discuss the need for data harmonization to improve disability research and policy. We describe standard question sets on disability developed for inclusion in surveys and administrative systems, as well as the need for coordination of both statistical and administrative data systems. Until disability data become more harmonized, it will not be possible to support the development of comprehensive, evidence-based policies and programs to address the needs of the population with disabilities.

Globally, people with disabilities have worse outcomes across a wide range of indicators, including poverty, employment, education, health, and violence.^1,2^ The Convention on the Rights of Persons with Disabilities, the driving force behind international efforts to promote the rights of people with disabilities, calls for data collection in support of ensuring those rights.^3^ The United Nations (UN) Sustainable Development Goals are based on the principle of “leave no one behind” and include a requirement for data collection to determine whether economic and social developments are benefiting all vulnerable groups.^4^ In the United States, the need for data to advance equity, including for people with disabilities, is recognized in executive order 13985 (“Executive Order on Advancing Racial Equity and Support for Underserved Communities through the Federal Government”).^5^ Although high-quality data collected on an ongoing basis are necessary to understand and reduce these gaps, the methodology for collecting data on disability across multiple sources is often inconsistent and, at times, flawed, not only across countries but even between data instruments within a single country.^6^ This creates confusion and undermines efforts to develop targeted policies and evaluate them appropriately. This article provides guidance on building an appropriate disability data system within a country to support policy efforts aimed at improving the lives of people with disabilities.

In this article we define *disability* as the result of the negative interaction between people who have difficulties in functioning in core activity domains and attitudinal and environmental barriers. People with disabilities are those who have functional limitations and therefore are at risk of exclusion when faced with these barriers. This definition is embedded in the question sets developed by the Washington Group on Disability Statistics, as described below.

Disability data serve many important purposes. First, they can describe the functional status of the population and subpopulations defined, for example, by sex; age; and type, degree, date of onset, and cause of disability. *Functional status* is defined as the extent of difficulties in carrying out core activities such as hearing, seeing, walking, cognition, and communication. Second, the identification of the population with disabilities can be used to disaggregate outcome indicators, such as poverty, to identify gaps in participation in social roles such as attending school, working, and civic and social participation between people with and without disabilities overall and for various subpopulations. The differences in levels of participation in these routine activities between those with and without disabilities are referred to as *disability gaps.* Both describing the functional status of the population and identifying disability gaps are essential for understanding where policy is needed and which populations to target.

The third purpose of data collection is to identify the barriers that create disability-related outcome gaps and the types of facilitators and supports that reduce or eliminate them. This can serve as the basis for designing and implementing policies and programs. In addition, data on the causes and precursors of functional limitations can be used for designing prevention programs.

The fourth purpose of data collection is to evaluate efforts to address gaps in outcome indicators between people with and without disabilities. This purpose can be met by combining data on the implementation of policies and programs with population data on disability status, environmental data on barriers and supports, and outcome indicators after the implementation of the policies and programs.

## Identifying People With Disabilities

According to the Convention on the Rights of Persons with Disabilities, “persons with disabilities include those who have long-term physical, mental, intellectual or sensory impairments which in interaction with various barriers may hinder their full and effective participation in society on an equal basis with others.”^7^ That is, disability is not located within the individual but rather emerges from the interaction between a person’s functional limitation and barriers in their environment that prevent fulfillment of social roles on an equal basis with others. This conception of disability drives prevention, medical care, and rehabilitation, but it also focuses on building a more inclusive environment. A person may be less likely to work not because they have functional difficulties but because they experience an environment that does not accommodate them—for example, with accessible buildings, transportation, and labor policies.

Once the population with disabilities is identified, it is possible to monitor their level of inclusion. Disparities can be tracked to determine whether policies to achieve full inclusion have been successful.

However, functional difficulties, and therefore disability, are not binary but lie along a continuum. Deciding on a cutpoint along that continuum of difficulty to define those with disabilities depends on the purpose for identifying that population. For example, the broadest measure of disability relates to civil rights legislation. This would include not only people who have functional difficulties but also those who have any impairment that may serve as the basis for discrimination, as found in the Americans with Disabilities Act of 1990. A more restrictive measure would include all people who would benefit from services and the removal of barriers related to their functional difficulties—for example, the receipt of support services in schools or vocational rehabilitation. An even more restrictive cutpoint would include people for whom such benefits and services are essential for maintaining their lives and livelihood. Data on the full spectrum of functional difficulties can help inform a more complete and nuanced approach to policy analysis.

## The Washington Group Question Set

To address the need for high-quality, comparable data, in 2001 the UN Statistical Commission established the Washington Group on Disability Statistics, comprising representatives from national statistical offices of UN member states and other partners. The group’s mandate is to develop high-quality, internationally comparable methods to identify people with disabilities and promote and coordinate international cooperation in generating and disseminating disability statistics.

The first task of the Washington Group was to develop a question set for inclusion in censuses. The Washington Group Short Set on Functioning (WG-SS) obtains information on the degree of difficulty in six basic functional domains: seeing, hearing, walking or climbing steps, remembering or concentrating, washing all over or dressing, and communicating.^8^ Domains were chosen to identify most people who have functional limitations that put them at risk of participation limitations. Each question has four response categories: no difficulty, some difficulty, a lot of difficulty, and cannot do at all. Disability status is defined by a response of “a lot of difficulty” or “cannot do at all” on at least one of the six questions.^9^ The WG-SS can also be used to identify subpopulations with varying degrees of difficulty across the different functional domains. Because it was developed for censuses, it had to be short and easy to administer.

The questions, adopted in 2006 after testing in a wide range of countries, are endorsed by both national and international organizations.^10,11^ According to a 2022 report to the UN Statistical Commission, between 2009 and 2021, 111 countries had included the WG-SS in a census or survey, with thirty-four additional countries intending to do so by 2022.^12^ The questions have been recommended by organizations of people with disabilities and international nongovernmental organizations, such as Save the Children, Global Action on Disability Network, Humanity and Inclusion, and Education Cannot Wait, as well as by international development agencies, including the Department of Foreign Affairs and Trade of Australia; the Foreign, Commonwealth, and Development Office of the United Kingdom; the Norwegian Agency for Development Cooperation; the US Agency for International Development; the World Bank Group; the German Agency for International Cooperation; the UN Statistical Division; and the UN Economic Commission for Europe, as well as by an Expert Group under the auspices of the UN Department of Economic and Social Affairs for disaggregating Sustainable Development Goals indicators by disability.^12^ The Washington Group on Disability Statistics question sets are included in a growing number of different types of collections and data sets.^13^ Adopting this common question set is a first step toward developing a data infrastructure for disability research and policy.

## Additional Disability Data Needs

In addition to data that monitor functioning and disaggregate outcomes by disability to identify disability gaps, additional data are needed for policy development.

For instance, the date of disability onset is needed. Functional limitations can arise at any time, and timing affects the impact of disability on a person’s life and the kinds of accommodations needed. For example, a child with a disability may need accommodations to ensure school attendance and the receipt of a high-quality education, an adult acquiring a disability may need accommodations related to employment, and an older person may need supports to engage in civic activities.

Once the population with disabilities is identified, it is possible to monitor their level of inclusion.

In addition, the development of inclusive policies requires environmental data—namely, information about the barriers and supports that impede or facilitate participation in social roles such as schooling, work, civic and social activities, and family life. The Washington Group on Disability Statistics and International Labor Organization Labor Force Survey Disability Module, for example, asks about the need, availability, and use of workplace accommodations and the reasons why people are not employed.^14^ Fiji’s Education Management Information System is one example of an administrative system that collects data identifying children with disabilities (using the Child Functioning Module [CFM], a tool developed by the United Nations Children’s Fund and the Washington Group) and data on schools’ physical infrastructure, materials, human resources, and accessible communication.^15^ The World Health Organization’s rapid Assistive Technology Assessment tool asks about technology-related support needs.^16^ Data collection can also expand beyond functional capacity to collect data on support needs, both human and technological, that can inform policies and programs.^17^

## Achieving Data Harmonization For Disability Identification

Disability data are collected using a variety of methods, through surveys and censuses and through administrative systems that produce data as part of program operations. Some of these administrative systems are for disability-specific programs (for example, for the distribution of disability benefits), and others are not (for example, administrative systems associated with the provision of education). In addition, disability information needs to be collected in systems that have historically not included such measures, including clinical intake forms and syndromic surveillance activities. Harmonizing the use of standard question sets in all types of data collection is essential for comparing across data sources and for improving the quality and utility of disability data in general. Because they avoid stigma and preconceptions about disability, questions that use a functional approach are more objective and precise and thus serve as a more effective bridge across data systems.

Harmonizing data on disability identification does not mean changing eligibility criteria or legal statutes related to disability.

When the methodology for identifying people with disabilities differs across data collections, it eliminates the ability to compare results. If different systems produce different prevalence estimates, characteristics of people with disabilities, or gaps in outcomes and access to services between people with and without disabilities, there is no way of knowing whether the observed differences are a function of actual differences or of methodological variations. A lack of understanding of the source of differences across systems in what appear to be the same statistics undermines the confidence in all estimates and thus limits the usefulness of these data.

The use of a common set of questions based on functioning to define the population with disabilities in different data collections allows for the resulting data sets to be used jointly, making them more powerful. Using multiple data sets also provides a better understanding of the experience of various subpopulations within the population with disabilities and thus supports policy and program development. The advantages are greater when data can be linked at the unit level (for example, person, household, or school). However, even if linkage is not possible, having a common core set of disability questions in all data sources allows for comparing the experiences of like populations interacting with different ministries or departments and different levels of government (for example, local, state, or national). In addition, it would provide for a clearer evaluation of how people with disabilities are served by various programs. Moreover, if a policy or program changed its disability eligibility criteria, the implications could be estimated. Overall, a more comprehensive picture of the population with disabilities and their characteristics, including access to and use of services, program outcomes, and the impact of policies and programs on inclusion, would be obtained.

As noted previously, the WG-SS has been endorsed by international organizations and adopted globally, allowing for international comparisons, and therefore is a good candidate for use as a standard question set.^12,13^ Standardization of questions related to other aspects of disability, such as age at onset or participation barriers and facilitators, is also important, but the most crucial need is for a core set to identify the population with disabilities.

The next step in harmonization is identifying all statistical information, censuses, and surveys that could inform policy on disability and have them include the core set of questions. The core set should necessarily be short to accommodate time and space restrictions. For example, because the WG-SS was designed for censuses, it contains the minimum number of questions needed to identify the population with disabilities. In the US, the WG-SS questions have been included in nationally representative health and health-related surveys, such as the National Health Interview Survey, the National Health and Nutrition Examination Survey, and the National Survey of Family Growth, as well as the Household Pulse Survey, designed to measure social and economic trends during the COVID-19 pandemic.

Other data collection platforms can expand the number of questions to improve data quality. The Washington Group on Disability Statistics also created the Extended Set on Functioning (WG-ES), which includes additional questions on psychosocial difficulties, upper body mobility, pain, fatigue, and information on the use of assistive devices.^9^ The WG-SS Enhanced expands on the Short Set by adding questions on upper body functioning and psychosocial difficulties for use when the full WG-ES cannot be included in a data collection.^9^ The shorter question set is a subset of the longer question set, allowing for cross-walking among data collections.

As the WG-ES underidentifies children with developmental difficulties and omits other domains of particular relevance to children, the Washington Group and United Nations Children’s Fund collaborated to create the CFM, which has been used in dozens of countries.^18^ The WG-SS Enhanced and WG-ES are used to obtain information on adults, and the CFM, on functioning in children. The WG-SS is a core set that can be used in any data collection, whereas the WG-ES and CFM can be used in surveys where more questions can be included and tailored to specific age groups.

Including one of the core sets does not mean that questions must be limited to that core, only that the core is similarly included in all systems. A health survey, for example, may want more detail on functioning, medical conditions related to the functional difficulties, and the age of onset of functional difficulties. Harmonization implies not that additional data should not be collected but that the core functioning questions be included on the survey in such a way that those other questions do not influence the responses to the core questions—for example, placing additional content after the core set.

Next, it is important to identify administrative data systems that currently collect data on disability and those that do not but do collect important outcome or environmental measures that could be disaggregated if disability questions were included. The core questions should then be added to these data systems. Existing questions do not need to be removed, although this should be considered if they are based on problematic methodologies. Including standard core question sets in administrative systems is the approach being undertaken in South Africa^19^ and is also being considered in Vietnam, Kosovo, and Zimbabwe. One example of harmonization is in Fiji, where the CFM has been incorporated in both the Education Management Information System and the Multiple Indicator Cluster Survey.^20–22^

Once this is accomplished, a set of disaggregation guidelines based on the use of a common set of cutpoints can be created so that comparisons by type and degree of disability can be standardized across different reports. The standard Washington Group on Disability Statistics cutpoint is having at least a lot of difficulty in at least one functional domain. Alternatively, a cutpoint of having some difficulty in at least one functional domain could also be used. This begins to get at the range of disability.

Harmonizing data on disability identification does not mean changing eligibility criteria or legal statutes related to disability. Those are policy decisions. The goal of data harmonization is simply to create a data system with a common approach and a core question set.

Disability is a complex concept, which requires data of different types and from different sources.

Such harmonization can expand beyond government data. Often organizations of people with disabilities and other civil-society organizations collect data, both for their internal operational purposes and to amass evidence for advocacy purposes. If aligned with the same common core set of questions, these data can be used in conjunction with official data, enhancing advocacy efforts. This also applies to efforts at collecting big data—for example, through social media and crowdsourced accessibility mapping.

## Challenges In Harmonization

Several challenges exist in harmonizing disability data. Different entities gather disability data for different purposes, and agreeing on a common strategy, given this diversity, may be challenging. One way to overcome this is to explain how a common approach to measuring disability can be used for different purposes by adjusting cutpoints and by adding additional data that are related to the specific institutional and statutory requirements often associated with policies and programs.

There are administrative costs associated with adding questions or changing data tools. These costs do not simply derive from changing questionnaire forms but involve electronic systems and syntax used in aggregating and reporting data. If questions are added to these instruments, respondent burden will increase. Reducing burden is an important concern, but doing so at the cost of data quality or comparability limits the usefulness of the resulting data. What may appear to be a reasonable way to reduce burden can have the opposite effect, by increasing the complexity of the questions. Depending on the data tool, this can also raise issues of self-disclosure. For some data tools—for example, systems relating to disability benefits, health services, or special education services—asking questions about functional difficulties may seem appropriate to respondents. For other data sets— for example, those related to disability employment quotas—respondents may feel less comfortable answering because it is unclear to them how the data will be used.

Any change to data collection methodology will affect the ability to monitor trends. This can be ameliorated by introducing a change while maintaining existing methods for a period of time to allow cross-walking between methods.

There is also the political risk of identifying problems in how current programs reach people with disabilities. Administrators might not be eager to collect data that could reveal inadequate or poor-quality service delivery. Acceptance of the new approach might not be accomplished quickly. However, any progress would have beneficial impacts on policy development and serve to demonstrate the usefulness of harmonization across all data systems.

As the data collection world is changing, it is important to include the appropriate disability data collection objectives in any new or evolving system.

## Conclusion

Disability is a complex concept, which requires data of different types and from different sources. This includes data on the characteristics of people with disabilities, the environmental barriers and supports that might affect their participation in society, programs and services available to address the impact of functional limitations at both the individual and the societal levels, and outcome indicators useful in monitoring well-being and the impact of policies and programs.

At the core of all of this analysis is the identification of who has disability and the type and degree of that disability. Data needed for this identification are collected through various statistical and administrative systems. To use these data to their fullest extent in a way that is informative for policy, avoids confusion, and is in line with modern conceptions of what is meant by *disability,* it is important to harmonize those systems by adopting a common core set of questions on functioning that can serve as a bridge between data sources. ■
